# Antipyrin

**Published:** 1887-04

**Authors:** H. E. Stroud

**Affiliations:** Colorado


### ANTIPYRIN.

BY H. E. STROUD, M. D., OF COLORADO.

Up to this time I have seen but little in my journals concerning this great antipyretic, and so concluded to give my experience with it in a few cases. I obtained an ounce while treating a case of typhoid fever, but convalescence was established before it came to hand. I did not have to wait long, however, before an opportunity to test its merits presented in the person of Mrs. W——, who had miscarried, a week before I saw her, at about the fourth month of gestation. They thought the membranes were retained, as there were frequent pains and a constant, but moderate, flow of bright blood. Examination showed the os contracted, and I was satisfied expulsive action could be stimulated by using the tampon, with less danger than to dilate and remove by force. Accordingly, the vagina was thoroughly packed, and, as I expected, stimulated pains. As soon as I had left the house the lady, who is very headstrong, withdrew the tampon directly contrary to my orders. The most frightful hemorrhage occurred. I was summoned hastily, and a fresh tampon, ergot and stimulants, barely saved life. She rallied and did nicely for a week, when symptoms of septic poisoning suddenly developed a violent chill, lasting over an hour. Agonizing pain,metritis and cellulitis ; the temperature jumped up to 105° ; pulse 140, and the delirium and restlessness all pointed to great danger. Counsel was asked for, but before help arrived I gave 30 grains of antipyrin, and, as I was anxious to see what it would do, I gave nothing else. In two hours I returned in company with counsel, and to my surprise found the temperature down to 101°, and the pulse 117. Profuse perspiration had occurred in less than an hour after the drug was given, and continued for several hours ; but for the pain the patient would have been quite comfortable.

It is not the object of this paper to detail the treatment in the case, except as relates to the fever ; suffice it to say that the most thorough treatment was brought to bear upon the local inflammation. It was found by repeatedly using the thermometer that the effect of the antipyrin lasted about eight hours, when a steady rise occurred. When 103° were reached 30 grains more were given, and profuse sweating and prompt reduction again occurred. This time the temperature sank a degree below the normal and the effect lasted nearly twelve hours ; but, after some days, it required larger doses. We would give 30 grains and two 15-grain doses an hour apart, but this amount never had to be increased, and never repeated oftener than twice in twenty-four hours, and some days one or two doses would suffice. During its administration it became necessary to use means to check the excessive sweating it occasioned, and 1-60-grain doses of atropia and elixir of vitriol moderated it, at least. The case was full of complications, and for three weeks it seemed a very doubtful case, and but for the antipyrine she must have died. During this period there were two distinct relapses ; violent chills, followed by high fevers ; and twice we tried other means to reduce it. The sedative reduced the pulse but not the fever ; quinine caused acute mania ; cold sponging came the nearest filling the bill, but did n't compare with antipyrin in promptness and efficacy, as it reduced the pulse in proportion to the decrease in temperature, but there was no evidence of lessened heat power.

During these anxious three weeks there was a great deal of nausea and vomiting, and when everything else would be ejected the antipyrin could always be given and was never ejected, and with the exception of two brief trials it was the sole means used to control the fever ; and I am glad to add, in conclusion, that the lady has recovered.

Since the above case, I have treated a case of rheumatic fever. When first called I found the temperature 104°. I gave 30 grains antipyrin at once ; in two hours it was down to 101°, then the salicylates were brought to bear. In this case no sweating followed its use, but fever was promptly reduced and but moderate diaphoresis occurred.

From these, and a few other cases, I conclude as follows : 1. Antipyrin is the most effectual antipyretic at our command. 2. Its use is nearly devoid of danger. 3. It has no visible effect on the stomach, bowels, kidneys or head. 4. It seems to reduce a fever by checking the processes of oxydation. 5. It has in itself no effect in removing the cause or shortening the course of continued fevers, but by preventing the hyperpyrexia, the acute fatty and granular cardiac degenerations, will not occur, and the nerve centres are saved shock—all of which favor the action of other remedies and recovery. Armed with antipyrin, I feel that I have an efficient remedy with which to fight the most common of pathological conditions, viz, fever.

